# Patterns and determinants of healthcare utilization and medication use before and during the COVID-19 crisis in Afghanistan, Bangladesh, and India

**DOI:** 10.1186/s12913-024-10789-4

**Published:** 2024-04-03

**Authors:** Md Tauhidul Islam, Mieghan Bruce, Khurshid Alam

**Affiliations:** 1 Murdoch Business School, Murdoch University, 6150 Perth, WA Australia; 2 School of Veterinary Medicine and Centre for Biosecurity and One Health, Harry Butler Institute, Murdoch University, 6150 Perth, WA Australia

**Keywords:** Healthcare utilization, Medication use, COVID-19, Afghanistan, Bangladesh, India

## Abstract

**Background:**

COVID-19 rapidly spread through South Asian countries and overwhelmed the health systems that were unprepared for such an outbreak. Evidence from high-income countries showed that COVID-19 impacted healthcare utilization, including medication use, but empirical evidence is lacking in South Asia. This study aimed to investigate the effect of COVID-19 on healthcare utilization and medication use in South Asia.

**Method:**

The current study used longitudinal data from the ‘Premise Health Service Disruption Survey’ 2020 and 2021. The countries of interest were limited to Afghanistan, Bangladesh, and India. In these surveys, data related to healthcare utilization and medication use were collected for three-time points; ‘Pre-COVID phase’, ‘Initial phase of COVID-19 outbreak’, and ‘One year of COVID-19 outbreak’. Generalized estimating equation (GEE) along with McNemar’s test, Kruskal–Wallis test and χ^2^ test were applied in this study following the conceptualization of Andersen’s healthcare utilization model.

**Result:**

The use of healthcare and medication was unevenly impacted by the COVID-19 epidemic in Afghanistan, Bangladesh, and India. Immediately after the COVID-19 outbreak, respondents in Bangladesh reported around four times higher incomplete healthcare utilization compared to pre-COVID phase. In contrast, respondents in Afghanistan reported lower incomplete utilization of healthcare in a similar context. In the post COVID-19 outbreak, non-adherence to medication use was significantly higher in Afghanistan (OR:1.7; 95%CI:1.6,1.9) and India (OR:1.3; 95%CI:1.1,1.7) compared to pre-COVID phase. Respondents of all three countries who sought assistance to manage non-communicable diseases (NCDs) had higher odds (Afghanistan: OR:1.5; 95%CI:1.3,1.8; Bangladesh: OR: 3.7; 95%CI:1.9,7.3; India: OR: 2.3; 95% CI: 1.4,3.6) of non-adherence to medication use after the COVID-19 outbreak compared to pre-COVID phase.

**Conclusion:**

The present study documented important evidence of the influence of COVID-19 epidemic on healthcare utilization and medication use in three countries of South Asia. Lessons learned from this study can feed into policy responses to the crisis and preparedness for future pandemics.

**Supplementary Information:**

The online version contains supplementary material available at 10.1186/s12913-024-10789-4.

## Background

A global health emergency was declared on 30 January 2020 due to COVID-19 [1]. The COVID-19 pandemic has had a far-reaching impact on various aspects of society. It has introduced lockdowns and disrupted gatherings, impacting social and cultural norms [2]. Economically, it has interrupted businesses and led to widespread unemployment [3]. On the political front, governments worldwide have faced challenges in crisis management, impacting their leadership and underscoring the need for international cooperation to address global health challenges [4]. The most immediate and prominent impact of the pandemic was on public health. This pandemic revealed weaknesses in healthcare systems, especially in low and middle-income countries, which struggled to cope with the crisis [5]. Even in high-income countries like the United States, hospitals cancelled non-emergency surgeries due to the surge in COVID-19 patients [6], facing financial challenges [7]. However, the impact was harsher in countries with weaker healthcare structures [8]. In Bangladesh, for example, there was just one Intensive Care Unit (ICU) bed per 100,000 people, causing critical patients to miss out on vital ICU care [9]. Conversely, South Korea, a high-income country, had 11 ICU beds per 100,000 people [9].

During the surge of COVID − 19 in South Asia, the majority of the hospitals and health facilities in every country were inundated with COVID-19 patients, making it impossible to provide quality treatment to patients with acute or chronic conditions [10]. Health service disruption was observed for every branch of healthcare, including maternal health [11], child health [12], reproductive health [13], mental health [14], immunization [15], and the management of non-communicable diseases (NCDs) [16]. Typically, health service disruptions are localized, occurring in the zones affected by conflicts [17, 18] or areas affected by natural disasters [19]. However, nearly all countries experienced disruptions this time as health systems shifted their focus to managing COVID-19 patients [20].

South Asian countries have historically failed to put into practice effective public health policy due to decades of chronic underinvestment in public health infrastructure throughout most of the region [21]. Frontline workers, including healthcare professionals and first responders were faced with shortages of essential personal protective equipment (PPE), risking their own safety. Additionally, the scarcity of resources like oxygen, diagnostic kit, and inadequate ICU facilities compounded their challenges during the pandemic [22]. These dedicated individuals also faced the complexity of navigating inconsistent government policies and evolving public health guidelines [23]. Moreover, they struggled to provide care for non-COVID-related diseases due to the overwhelming number of COVID-19 cases. While e-health and m-health systems are well-established in high-income countries to provide care, South Asian nations had to develop these systems from scratch during the pandemic to address non-COVID-19 related health needs [24, 25]. For example, prior to the emergence of the COVID-19 pandemic, India had not implemented telemedicine extensively, and initial efforts had encountered obstacles. Moreover, the legal framework for telemedicine was not well-defined until the issuance of guidelines on March 25, 2020 [26].

Recent evidence indicated that COVID-19 pandemic significantly impacted utilization of healthcare in South Asia. Such as, in a tertiary hospital of India, 25.0% of patients during the lockdown phase and 17.4% of patients in the pre-lockdown phase arrived for medical attention with cardiovascular emergencies after the 12-hour window from the onset of symptoms had passed. This increase was significant compared to the pre-COVID period, where only 6% of patients presented with cardiovascular emergencies beyond the 12-hour window. Similarly, a tertiary hospital in Bangladesh observed a reduction of over 50% in acute stroke admissions during the COVID-19 pandemic [27]. The decline in health service utilization during the COVID-19 pandemic was influenced not only by factors related to the availability of healthcare services but also by factors associated with patient demand [28]. However, two systematic reviews on health service utilization patterns in the South Asian region during the COVID-19 pandemic highlighted a notable prevalence of cross-sectional and retrospective studies, often conducted at individual healthcare facilities [29, 30]. These studies predominantly relied on administrative data sources like hospital records and health management information systems (HMIS) [29, 30]. Importantly, very few studies have delved into the underlying factors contributing to the reduced utilization of health services during COVID-19, particularly using panel data and advanced statistical analysis techniques. Furthermore, it is noteworthy that the majority of these limited studies were conducted outside of South Asia [31–34]. Moreover, it is worth noting that very few studies distinguished between the impacts of supply-side factors (such as healthcare facility capacity and resource availability) and demand-side factors (including patient behaviours and preferences) on health service utilization [31–34]. It is imperative to acknowledge that health facilities in South Asia serve as major sources of medication and the scarcity of medicines was observed during the pandemic [35]. It is essential to recognize that without access to necessary medications, individuals may face significant challenges in controlling and halting the progression of diseases. However, it is noteworthy that the literature on this topic remains scarce in the South Asian context. Recent systematic reviews in this region identified only six studies out of 43 that specifically focused on access to medicine during the COVID-19 pandemic, and notably, none of them were conducted in Bangladesh or Afghanistan [29, 30].

Therefore, this study was undertaken to describe the changes in health service delivery in Afghanistan, Bangladesh, and India across three time periods: before COVID-19 outbreak, immediately after the COVID-19 outbreak, and one year after the start of the COVID-19 outbreak. This study adopted the World Bank’s South Asian region classification, encompassing eight countries, which include Afghanistan, Bangladesh, and India [36]. Intention was to include all eight countries of the South Asian region. However, during the planning and execution of our research, the study encountered limitations primarily related to data availability. Health service delivery was examined by observing healthcare utilization and medication use. The present study additionally observed the changes by supply-side factors and demand-side factors of health service disruptions separately. The study also investigated the factors associated with these changes.

## Method

### Data source

**Fig. 1:**
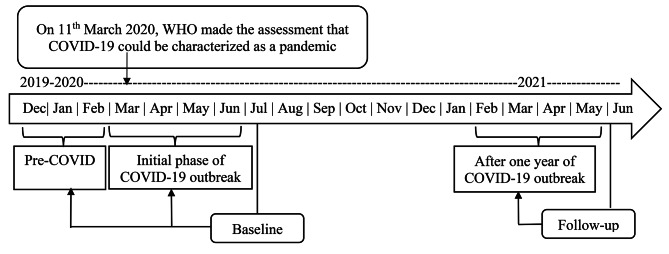
Premise health service disruption survey 2020 and 2021

Longitudinal data were drawn from the ‘Premise General Population COVID-19 Health Services Disruption Survey’ [37], designed to evaluate the interference with the delivery of general healthcare services, including access to healthcare professionals and prescription drugs caused by the COVID-19 pandemic and subsequent government directives and behavioural modifications to slow the spread of the disease. Thispanel survey was first fielded in July 2020, and the follow-up survey was conducted throughout June 2021. In the first survey, the data were collected for two time periods, from December 2019 to February 2020 and from March 2020 to June 2020. In the follow-up survey, data were collected for the period from February 2021 to May 2021 to give further insight into the changes in health service delivery throughout a specified period of the COVID-19 global pandemic (Fig. 1). For this study, the 1st time period, 2nd time period and 3rd time period were labelled as ‘Pre COVID-19 phase’, ‘Initial phase of COVID-19 outbreak’, and ‘One year of COVID-19 outbreak’, respectively. This survey was designed collaboratively by the Institute for Health Metrics and Evaluation (IHME), the University of Washington, and Bill and Melinda Gates Foundation (BMGF) and implemented by Premise, a smart phone based application that supports data collection around the world. There were no inclusion criteria other than the country of residence since this survey was designed to observe the health service delivery among the general population [37].

The current investigation included data from Afghanistan, Bangladesh, and India since the focus of this study was to examine South Asian countries only. In total, 5,026 participants from Afghanistan, Bangladesh, and India participated in the “Premise General Population COVID-19 Health Services Disruption Survey” in 2020. Those interviewed in 2020 also participated in the “Premise General Population COVID-19 Health Services Disruption Survey” in 2021, with 1669 participants completing both surveys [37].

### Measures

**Fig. 2:**
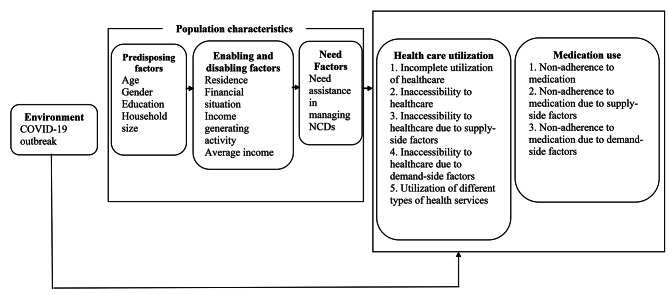
Conceptual framework of the study using Andersen’s model of health care utilization

Andersen’s model of health care utilization [38] was used to categorize the study variables into outcome variables, external environment factors, predisposing factors, enabling and disabling factors, and need for care factors (Fig. 2).

#### Outcome variables

**‘Incomplete utilization of the healthcare’** was assessed as ‘Yes’ if the participants expressed the need to utilize healthcare but did not utilize it for any reason. **‘Inaccessibility to healthcare’** was defined as ‘Yes’ if the participant responded ‘No’ and ‘I saw a provider during this time, but not every time I needed one’ to the question ‘Were you able to see a health provider?’ When participants were asked to provide the reasons for inaccessibility, the response options were ‘Health facility closed’, ‘Turned away from health facility’, ‘Treatment or tests unavailable’, ‘No transportation’, ‘Lack of money’ ‘Partner or family does not approve’, ‘Fear of contracting COVID-19 infection’, ‘Other’ and ‘Decline to respond’. This study divided these reasons into ‘supply-side factors’ and ‘demand-side factors’. ‘Health facility closed’, ‘Turned away from health facility’, and ‘Treatment or tests unavailable’ options were categorized as ‘supply-side factors’, and the rest of the options were categorized as ‘demand-side factors’. Both of the factors were considered as a separate outcome and named as **‘Inaccessibility to healthcare due to supply-side factors’** and **‘Inaccessibility to healthcare due to demand-side factors’. ‘Non-adherence to medication’** was considered ‘Yes’ if the participant missed a dose of medication. Later, they were asked for the reasons of non-adherence to medication. For study purpose, reasons ‘Pharmacy closed’, ‘Turned away from pharmacy’, ‘Medication unavailable at pharmacy’ were categorized as ‘supply-side factors’. Further reasons like ‘No transportation’, ‘Lack of money’, ‘Partner or family does not approve’, ‘Fear of contracting COVID-19 infection’, ‘Forget to take medicine’, ‘Other’, and ‘Decline to respond’ were considered ‘demand-side factors’. These two derived variables were named **‘Non-adherence to medication due to demand-side factors’** and **‘Non-adherence to medication due to supply-side factors’.** Unlike other outcome variables, **‘Utilization of different types of health services’** was collected for only two time periods, from the ‘Pre-COVID phase to Initial phase of COVID-19 outbreak’ to ‘After one year of COVID-19 outbreak’, and changes in utilization were observed separately for ‘In-person healthcare’, and ‘Non-conventional care, which includes telemedicine, care at home and others.

### Explanatory variables

Experience of participants in three different periods was assessed to see the effect of COVID-19 outbreak and considered as a separate explanatory variable under external environment factors. Participants’ ages were divided into two categories: ‘Less than 26 years’ and ’26 years and above’. One primary factor influencing the choice of this age category was the well-recognized variation in health-seeking behaviour within this age group [39]. Additionally, there might be variations in the prevalence of both supply-side and demand-side factors across this age group that might also impact healthcare access. For instance, younger individuals in this group may had lower levels of COVID-19-related fear, potentially made them less likely to alter their healthcare utilization patterns. Residence of the participants were characterised as urban and rural. Less than tertiary education refers to those participants who did not enrol into a university or less. ‘Large household’ refers to that household where the number of household members is five or more. ‘Poor financial situation’ refers to those who responded that they could only afford food for their family or worse than that. The rest of the respondents were categorized as ‘Better financial situation’. Income of the participants was collected as the average income in a month. Participants were also categorized based on their participation in income-generating activity. Need assistance in managing NCDs refers to those participants who expressed the need to visit a health care provider or purchase medicines for the management of NCDs. Heart disease, high blood pressure, stroke, cancer, diabetes, high blood cholesterol, asthma, mental health, injury, hearing or vision problems, alcohol or drug problems, and orthopaedic disorders (other than traumatic injuries) were considered NCDs. All the variables mentioned above were time-variant factors. Among those, income, participation in income-generating activity and need assistance in managing NCDs were collected for three time periods, and the rest of the variables were collected for two time periods.

### Statistical analysis

The baseline characteristics and characteristics of the participants who participated in the survey after one year of COVID-19 outbreak were represented as proportions by countries with 95% confidence interval (95% CI) for categorical variables. Given the skewness of income variable, it was presented by countries with median and interquartile range (IQR). There were several outliers in income variable which were treated by winsorization [40], where extreme values were replaced by 95% percentile of income, which is not an outlier. Log conversion was done for income before the formal analysis. Currency conversion was done from local currency to USD using the mid-time point currency exchange rate using www.xe.com [41]. Comparisons across three countries were conducted using Kruskal–Wallis test for continuous variables and χ^2^ test for categorical variables. McNemar’s test for outcome variables was used to compare the difference between ‘Pre COVID-19 phase’, ‘Initial phase of COVID-19 outbreak’, and ‘One year of COVID-19 outbreak’ within matched pairs. Further, considering the complex nature of data, the associations between the repeated measures of outcome variables and different factors were tested for statistical significance by fitting regression models to the data for each outcome variable using a Generalised Estimating Equation (GEE) approach. The GEE model was fitted with a logit link function and binomial family with exchangeable working correlation structures. The GEE approach pooled the repeated measures on each explanatory variable and on each outcome variable at ‘Pre COVID-19 phase’, ‘Initial phase of COVID-19 outbreak’, and ‘One year of COVID-19 outbreak’ to produce an estimate of the population-averaged association between different time periods and the outcome variables. For the dichotomous measures a logistic regression model was fitted of the form:

$$ logit\left({Y}_{it}\right)={B}_{0}+{B}_{1}{X}_{it}$$

Here, the logit (Y_it_) represents the log odds of the outcome. Y for the i-th respondent during different time periods: ‘Pre COVID-19 phase’, ‘Initial phase of COVID-19 outbreak’, and ‘One year of COVID-19 outbreak’. Additionally, we expanded the GEE models to incorporate a set of covariate factors outlined in the Anderson model (Fig. 2).

For dichotomous outcome the following model was used.

$$ logit\left({Y}_{it}\right)={B}_{0}+{B}_{1}{X}_{it}+\sum {B}_{m}{Z}_{imt}+ {B}_{n}{Z}_{in}$$

Here, Z_imt_ refers to dynamic covariates over time, and Z_in_ is a fixed covariate representing an individual’s gender. The coefficient B_1_ in the models indicates the impact of different time periods on the outcome, considering the effects of other covariates. To assess the strength of association between outcome variables and explanatory variables, crude, and adjusted odds ratio (OR) with a 95% CI were computed. Variables having less than 5% *p* value in the multivariable GEE model were considered significantly associated factors with the outcome variables. The results were weighed using the survey weights calculated by the ‘Premise General Population COVID-19 Health Services Disruption Survey’ to mitigate the effects of nonrepresentative samples [37]. All analyses were conducted using the STATA version 17 (basic edition).

### Ethical considerations

The data from Premise COVID-19 Health Services Disruption Survey 2020 and 2021 are archived in the IHME, which is freely available for research purposes. There is no information from which a respondent can be identified from the dataset [37]. The study proposal underwent a thorough review by the Human Ethics Committee of Murdoch University, and an ethics exemption was granted (Protocol number 2022/123).

## Results

### Characteristics of the participants

Table 1 shows the baseline characteristics of the participants. A higher representation (more than 75%) of male participants was observed in this survey for all three countries. Number of participants were higher in the urban areas compared to the rural areas in all three countries. Around 67% of participants in Afghanistan and 69% in Bangladesh faced poor financial situations compared to 45.5% in India at the baseline. During the ‘Pre-COVID phase’, 36.5% of participants in Bangladesh did not participate in any income-generating activity, which went up to 48.0% for the period of the ‘Initial phase of COVID-19 outbreak’. On the other hand, the opposite trend was observed in Afghanistan (49.1% vs. 36.4%) and in India (47.6% vs. 42.8%) in regard to income-generating activity. In the ‘Pre COVID-19 phase’, the median average income in a month for participants in Bangladesh and India was USD 71.8 and USD 142, respectively. However, during the ‘Initial phase of COVID-19 outbreak’, these figures dropped significantly to USD 48 and USD 67.3 for Bangladesh and India, respectively. In contrast, Afghan participants saw a slight decrease in their median average income from USD 13.8 USD in the ‘Pre COVID-19 phase’ to USD 11.5 during the ‘Initial phase of COVID-19 outbreak’. About 75.5%, 68.1%, and 67.9% of participants from Afghanistan, Bangladesh, and India, respectively needed health services for NCD management. Characteristics of the participants who participated in the survey after one year of COVID-19 outbreak are presented in Supplemental Table 1.

**Table 1:** Baseline characteristics of the participants

Characteristics	Afghanistan^†^ *n* = 3836	Bangladesh^‡^ *n* = 200	India^***^ n = 990
	Percentage (95% CI)/ Median (IQR)	Percentage (95% CI)/ Median (IQR)	Percentage (95% CI)/ Median (IQR)
**Predisposing factors**			
Age*			
Less than 26 years	77.7 (76.3, 79.0)	64.1 (57.1, 70.6)	68.8 (65.8, 71.6)
26 years and above	22.3 (21.0, 23.7)	35.9 (29.4, 42.9)	31.2 (28.3, 34.2)
Gender*			
Male	86.3 (85.1, 87.5)	92.0 (87.0, 95.2)	74.1 (71.2, 76.8)
Female	13.7 (12.5, 14.8)	8.0 (4.8, 12.7)	25.9 (23.2, 28.8)
Education*			
Less than tertiary education	56.0 (54.3, 57.5)	29.4 (23.3, 36.3)	37.8 (34.8, 40.9)
Tertiary education or more	44.0 (42.2, 45.6)	70.6 (63.7, 76.6)	62.2 (59.0, 65.2)
Household size*			
Large household	48.9 (47.2, 50.5)	42.7 (36.0, 49.7)	31.8 (28.9, 34.8)
Small household	51.1 (49.4, 52.7)	57.3 (50.2, 63.9)	68.2 (65.2, 71.0)
**Enabling/Disabling factors**			
Residence*			
Rural	34.4 (32.9, 35.9)	37.5 (31.0, 44.4)	41.9 (38.8, 45.0)
Urban	65.6 (64.0, 67.1)	62.5 (55.6, 69.0)	58.1 (55.0, 61.2)
Financial situation*			
Poor financial situation	66.9 (65.3, 68.4)	68.8 (61.9, 74.8)	45.5 (42.4, 48.6)
Better financial situation	33.1 (31.6, 34.6)	31.2 (25.1, 38.0)	54.5 (51.3, 57.6)
Income generating activity during ‘Pre-COVID phase’ *			
No	49.1 (47.4, 50.6)	35.7 (29.4, 42.6)	47.9 (44.8, 51.0)
Yes	50.9 (49.3, 52.5)	64.3 (57.3, 70.6)	52.1 (48.9, 55.2)
Income generating activity during ‘Initial phase of COVID-19 outbreak’ *			
No	36.4 (34.8, 37.9)	48.0 (41.0, 54.9)	42.8 (39.7, 45.9)
Yes	63.6 (62.0, 65.1)	52.0 (45.0, 58.9)	57.2 (54.0, 60.2)
Average income (USD) in a month during ‘Pre-COVID phase’*	13.8 (84.4)	71.8 (165.2)	142.0 (345.4)
Average income (USD) in a month during ‘Initial phase of COVID-19 outbreak’ *	11.5 (78.9)	48.0 (116.5)	67.3 (238.5)
**Need for care factors**			
Need assistance in managing NCDs during ‘Pre-COVID phase’ *			
Yes	75.5 (73.8, 76.9)	68.1 (60.1, 75.2)	67.9 (63.9, 71.6)
No	24.5 (23.0, 26.1)	31.9 (24.8, 39.9)	32.1 (28.4, 36.1)
Need assistance in managing NCDs with during ‘Initial phase of COVID-19 outbreak’ *			
Yes	74.9 (73.3, 76.5)	67.5 (59.3, 74.8)	68.6 (64.4, 72.4)
No	25.1 (23.5, 26.7)	32.5 (25.2, 40.7)	31.4 (27.5, 35.5)

^†^ Number of missing values for Afghanistan: Gender = 144, Age = 12, Household size = 192, Education = 64

^‡^ Number of missing values for Bangladesh: Gender = 2, Education = 4

^***^ Number of missing values for India: Gender = 15, Household size = 1, Education = 13

*All analyses were weighted. All the variables are significantly different (*p* value < 0.05)

Note: Comparisons across three countries were conducted using Kruskal–Wallis test for continuous variables and χ^2^ test for categorical variables

Abbreviations: CI, Confidence Interval; IQR, Interquartile Range; USD, United States Dollar; NCDs, Non-communicable diseases

### Change in healthcare utilization in Afghanistan, Bangladesh, and India

**Fig. 3:**
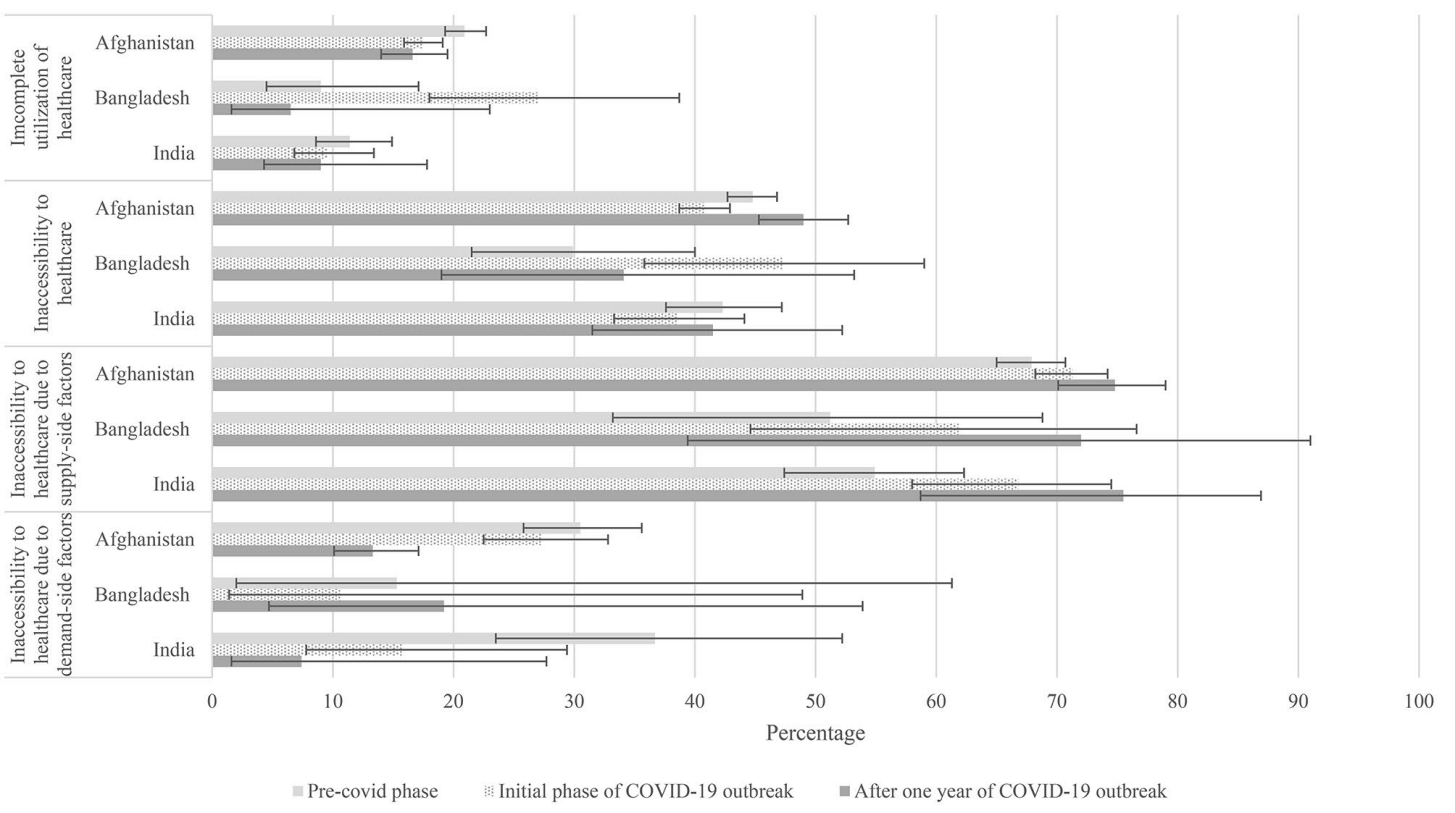
Distribution of healthcare utilization characteristics in Afghanistan, Bangladesh, and India

Figure 3 shows the healthcare utilization characteristics at three different time periods for Afghanistan, Bangladesh, and India, and the change from the ‘Pre-COVID phase’ to the ‘Initial phase of COVID-19 outbreak’ and the ‘After one year of COVID-19 outbreak’. The statistical significance of these changes was tested and is presented in Supplemental Table 2. About 20.9% of participants in Afghanistan did not utilize health care despite their needs. In this aspect, a downward trend was observed in the following period, and these changes were statistically significant (*p* value < 0.05). A similar trend was observed for India as well, but these changes were not statistically significant at 5% level. Nearly 27.1% of participants in Bangladesh experienced incomplete utilization of healthcare during the ‘Initial phase of COVID-19 outbreak’, while 6.5% and 9.0% of respondents experienced incomplete utilization of healthcare during the ‘Pre-COVID phase’ and the ‘After one year of COVID-19 outbreak’, respectively. During the ‘Initial phase of COVID-19 outbreak’, 47.3% of respondents in Bangladesh faced healthcare inaccessibility, which was significantly different from the ‘Pre-COVID phase’ (34.1%) with a *p* value less than 0.05. Unlike Bangladesh, in Afghanistan, the percentage of inaccessibility to healthcare during the ‘Initial phase of COVID-19 outbreak’ (40.8%) was less than the percentage of inaccessibility to healthcare during the ‘Pre-COVID phase’ (44.8%), and it is statistically significant (*p* value < 0.05). The majority of the participants in Afghanistan, Bangladesh, and India experienced difficulty in getting services from healthcare due to supply-side factors, and it increased over three time periods, although these changes were not always statistically significant. In contrast, inaccessibility to healthcare as a result of demand-side factors went down over the three-time periods for all three countries. The statistically substantial shift was observed for Afghanistan only.

### Medication use and its change in Afghanistan, Bangladesh, and India

**Fig. 4:**
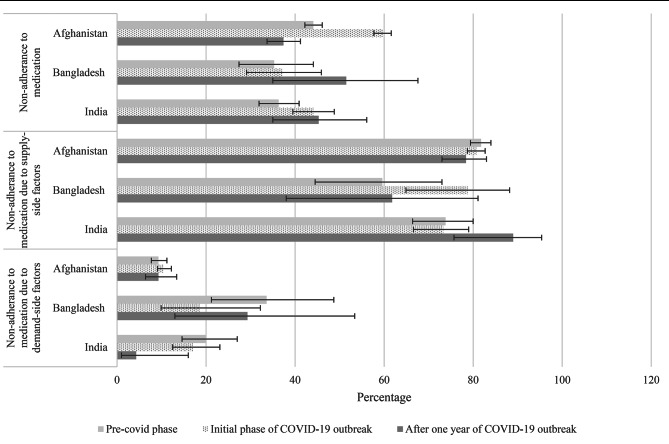
Distribution of medication use characteristics in Afghanistan, Bangladesh, and India

Figure 4 illustrates the medication use at three different time periods for Afghanistan, Bangladesh, and India and it’s change from the ‘Pre-COVID phase’ to the ‘Initial phase of COVID-19 outbreak’ and the ‘After one year of COVID-19 outbreak’. Supplemental Table 2 provides a detailed account of the analysis used to assess the significance of these changes. During the ‘Initial phase of COVID-19 outbreak’ in Afghanistan, approximately 60.0% of participants experienced non-adherence to medication. This percentage differed significantly from the ‘Pre-COVID phase’ (44.1%) and the ‘One year after COVID-19 outbreak’ (37.4%), and these variations were statistically significant (*p* value < 0.05). In India during the ‘Pre-COVID phase’ 36.3% of respondents encountered missing doses of medication, which increased to 44.1%, and the change is statistically significant (*p* value < 0.05). In this regard, no significant variation was observed for Bangladesh. However, in Bangladesh around 50% of the participants experienced non-adherence to medication during the ‘One year after COVID-19 outbreak’, which was almost 15% higher than the ‘Pre-COVID phase’. Here, reasons for non-adherence to medication were also examined from the angle of supply-side and demand-side factors, where many of the participants of these three countries reported supply-side factors for being non-adherent to medication. But any notable change was not observed, except for Afghanistan. Around 20.1% of participants in Afghanistan mentioned demand-side factors as their reason for non-adherence to medication during the ‘Pre-COVID phase’, which reduced to 17.1% during ‘Initial phase of COVID-19 outbreak’, and this change was statistically significant (*p* value < 0.05) (supplemental Table 2).

### Adjusted association between outcome variables (healthcare utilization and medication use) and explanatory variables

#### Afghanistan

The adjusted model (Table 2) showed that during the ‘Initial phase of COVID-19 outbreak’ respondents of Afghanistan were 30% less likely to experience incomplete utilization of healthcare (OR:0.7; 95%CI:0.6,0.9), 20% less likely to experience inaccessibility to healthcare (OR:0.8; 95%CI:0.8,0.9) and non-adherence to medication due to supply-side factors (OR:0.8; 95%CI:0.7,0.9) compared to the ‘Pre-COVID phase’. During the same period, the respondents were 70% more likely to experience non-adherence to medication (OR:1.7; 95%CI:1.6,1.9) and 40% more likely to non-adherence to medication due to demand-side factors (OR: 1.4; 95%CI:1.1,1.7). After one year of COVID-19 outbreak, the participants were 50% more likely to face difficulty in accessing healthcare due to supply-side factors (OR:1.5; 95%CI:1.1,2.0) compared to the ‘Pre-COVID phase’. On the other hand, one year following the COVID-19 outbreak, there was a 30–80% decrease in the likelihood of healthcare inaccessibility due to demand-side factors (OR:0.2; 95%CI: 0.1,0.4), non-adherence to medication (OR:0.7; 95%CI: 0.6,0.9), and non-adherence to medication due to supply-side factors (OR:0.7; 95%CI: 0.5,0.9).

**Table 2:** Multivariate generalized estimating equation model^†^ to examine the association between the predictor variables and healthcare utilization, and use of medication

Variable	Incomplete utilization of healthcare	Inaccessibility to health care	Inaccessibility to healthcare due to supply-side factors	Inaccessibility to healthcare due to demand-side factors	Non-adherence to medication	Non-adherence to medication due to supply-side factors	Non-adherence to medication due to demand-side factors
	OR (95% CI)	OR (95% CI)	OR (95% CI)	OR (95% CI)	OR (95% CI)	OR (95% CI)	OR (95% CI)
**Afghanistan**							
**Environmental factor**							
Initial phase of COVID-19 outbreak	0.7 (0.6,0.9)*	0.8 (0.8,0.9)*	1.0 (0.8,1.2)	0.9 (0.7,1.1)	1.7 (1.6,1.9)*	0.8 (0.7,0.9)*	1.4 (1.1,1.7)*
After one year of COVID-19 outbreak	0.9 (0.7,1.1)	1.1 (0.9,1.3)	1.5 (1.1,2.0)*	0.2 (0.1,0.4)*	0.7 (0.6,0.9)*	0.7 (0.5,0.9)*	1.1 (0.7,1.7)
**Pre-disposing factors**							
26 years and above	1.0 (0.8,1.3)	1.0 (0.8,1.2)	0.8 (0.6,1.0)	1.1 (0.8,3.4)	0.7 (0.6,0.9)*	0.9 (0.7,1.2)	1.07 (0.7,1.5 )
Male	0.8 (0.6,1.1)	0.8 (0.6,1.0)	1.3 (0.9,1.8)	0.7 (0.5,1.0)	1.1 (0.9,1.4)	1.3 (0.9,1.9)	0.6 (0.3,1.02)
Less than tertiary education	0.9 (0.7,1.1)	1.1 (0.9,1.2)	1.2 (1.02,1.5)*	0.8 (0.6,1.0)	1.1 (0.9,1.2)	1.3 (1.1,1.7)	0.7 (0.5,1.07)
Large household	0.8 (0.7,1.0)	0.4 (0.4,0.5)*	0.4 (0.3,0.5)*	2.4 (1.9,3.0)*	0.9 (0.7,1.0)	0.3 (0.2,0.4)*	4.0 (2.8,5.9)*
**Enabling/disabling factors**							
Rural	0.8 (0.6,0.9)*	0.7 (0.6,0.8)*	0.9 (0.7,1.1)	1.0 (0.8,1.3)	1.4 (1.2,1.6)*	1.0 (0.8,1.3)	0.7 (0.5,1.0)
Poor financial status	0.8 (0.7,1.0)	0.8 (0.7,1.0)	1.2 (1.02,1.5)*	0.8 (0.6,1.0)	0.9 (0.8,1.1)	1.3 (1.02,1.6)*	0.8 (0.6,1.1)
Not participating in an income-generating activity	2.1 (1.8,2.5)*	1.8 (1.6,2.1)*	0.9 (0.7,1.1)	1.1 (0.8,1.3)	0.4 (0.4,0.5)*	0.5 (0.4,0.7)*	1.7 (1.3,2.2)
Income	0.9 (0.9,0.9)*	0.9 (0.9,0.9)*	0.9 (0.9,0.9)*	1.0 (1.0,1.09)	0.9 (0.9,1.0)	0.9 (0.9,1.0)	1.0 (0.9,1.0)
**Need for care factor**							
Need assistance in managing NCDs	0.9 (0.7,1.1)	1.1 (0.9,1.3)	1.2 (1.01,1.6)*	0.7 (0.5,0.8)*	1.5 (1.3,1.8)*	1.4 (1.07,1.9)*	0.3 (0.2,0.4)*
**Bangladesh**							
**Environmental factor**							
Initial phase of COVID-19 outbreak	3.7 (1.4,9.4)*	2.0 (1.1,3.7)*	1.1 (0.4,3.0)	0.8 (0.3,2.1)	1.1 (0.8,1.7)	2.3 (1.3,4.1)*	0.4 (0.3,0.8)*
After one year of COVID-19 outbreak	1.1 (0.2,5.5)	1.4 (0.5,3.9)	2.8 (0.6,12.5)	0.1 (0.03,0.6)*	1.5 (0.6,3.7)	0.5 (0.1,1.7)	1.7 (0.5,5.6)
**Pre-disposing factors**							
26 years and above	2.5 (1.05,6.2)*	1.8 (0.8,3.9)	0.2 (0.06,1.3)	3.5 (0.7,17.5)	1.5 (0.5,2.1)	0.9 (0.3,2.5)	1.1 (0.3,3.4)
Male	0.4 (0.06,2.5)	0.7 (0.2,2.5)	-	-	0.8 (0.2,2.5)	1.3 (0.2,6.1)	3.1 (0.4,21.1)
Less than tertiary education	0.8 (0.3,2.2)	0.8 (0.3,1.9)	2.5 (0.6,9.3)	0.4 (0.1,1.7)	1.0 (0.5,2.1)	0.4 (0.1,1.7)	1.9 (0.5,6.9)
Large household	1.3 (0.5,3.6)	1.2 (0.6,2.5)	0.9 (0.2,3.4)	1.1 (0.3,4.3)	0.9 (0.4,1.7)	0.2 (0.09,0.7)*	3.0 (1.1,8.4)*
**Enabling/disabling factors**							
Rural	0.4 (0.1,1.2)	0.5 (0.2,1.1)	0.4 (0.1,1.5)	2.0 (0.5,7.8)	1.3 (0.6,2.5)	1.6 (0.6,4.7)	0.7 (0.2,2.2)
Poor financial status	2.6 (0.8,8.2)	1.4 (0.6,3.3)	0.6 (0.1,3.5)	1.3 (0.2,7.8)	0.6 (0.2,1.4)	2.7 (1.003,7.5)*	0.3 (0.1,0.8)*
Not participating in an income-generating activity	1.1 (0.4,2.9)	1.5 (0.7,3.2)	0.6 (0.2,2.2)	1.8 (0.5,6.3)	0.6 (0.3,1.0)	0.6 (0.2,1.6)	0.9 (0.3,2.4)
Income	1.1 (0.9,1.4)	0.9 (0.7,1.0)	0.8 (0.6,1.1)	0.3 (0.09,1.3)	1.0 (0.8,1.1)	1.1 (0.9,1.5)	0.8 (0.7,1.1)
**Need for care factor**							
Need assistance in managing NCDs	0.7 (0.2,2.0)	1.3 (0.6,2.9)	2.1 (0.5,7.5)	0.3 (0.09,1.3)	3.7 (1.9,7.3)*	3.4 (0.8,14.1)	0.1 (0.04,0.8)*
**India**							
**Environmental factor**							
Initial phase of COVID-19 outbreak	0.8 (0.5,1.3)	0.9 (0.6,1.1)	1.4 (0.8,2.4)	0.6 (0.4,1.1)	1.3 (1.1,1.7)*	0.9 (0.6, 1.4)	0.9 (0.6,1.3)
After one year of COVID-19 outbreak	0.6 (0.2,1.5)	0.8 (0.4,1.3)	1.5 (0.6,3.6)	0.09 (0.02,0.4)*	1.0 (0.5,1.6)	2.7 (1.1,6.5)*	0.1 (0.06,0.3)*
**Pre-disposing factors**							
26 years and above	0.9 (0.5,1.6)	0.8 (0.6,1.2)	1.1 (0.6,1.9)	0.8 (0.4,1.4)	1.2 (0.8,1.9)	0.3 (0.1,0.6)*	2.3 (1.1,4.8)*
Male	1.2 (0.6,2.2)	1.0 (0.7,1.6)	1.0 (0.6,1.9)	0.8 (0.4,1.4)	1.1 (0.7,1.8)	1.3 (0.5,2.8)	0.6 (0.2,1.6)
Less than tertiary education	1.3 (0.7,2.1)	1.1 (0.7,1.6)	1.1 (0.6,1.8)	0.8 (0.5,1.3)	1.2 (0.8,1.7)	0.9 (0.4,1.6)	1.4 (0.7,2.8)
Large household	0.7 (0.4,1.2)	0.6 (0.4,0.9)*	0.5 (0.2,0.9)*	1.8 (1.07,3.2)*	0.7 (0.4,1.0)	0.3 (0.1,0.6)*	2.6 (1.3,5.2)*
**Enabling/disabling factors**							
Rural	1.0 (0.6,1.7)	1.0 (0.7,1.4)	1.4 (0.8,2.4)	0.7 (0.4,1.1)	1.6 (1.1,2.3)*	1.1 (0.6,2.1)	0.8 (0.4,1.6)
Poor financial status	1.4 (0.8,2.4)	1.0 (0.7,1.3)	0.6 (0.3,1.02)	1.6 (0.9,2.7)	1.4 (1.02,2.0)*	0.9 (0.4,1.7)	1.1 (0.5,2.4)
Not participating in an income-generating activity	2.0 (1.2,3.5)*	1.7 (1.2,2.4)*	0.8 (0.4,1.3)	1.1 (0.6,1.9)	0.3 (0.2,0.4)*	0.3 (0.1,0.6)*	2.2 (1.1,4.3)*
Income	0.9 (0.8,1.0)	1.0 (0.9,1.0)	0.9 (0.8,1.0)	1.0 (0.9,1.1)	1.0 (0.9,1.0)	0.9 (0.8,1.0)	0.9 (0.9,1.0)
**Need for care factor**							
Need assistance in managing NCDs	1.2 (0.7,2.1)	1.0 (0.7,1.5)	1.1 (0.6,1.9)	0.8 (0.4,1.4)	2.3 (1.4,3.6)*	2.3 (1.1,4.6)*	0.3 (0.1,0.7)*

* *p* value < 0.05

^†^All covariates were mutually adjusted

Reference group for Impact of COVID = “Pre-COVID phase”, gender = female, age = less than 26 years, education = less than tertiary education, household size = Small household, residence = urban, financial status = better financial status, income generating activity = participated in income-generating activity, and need for care factors = those who sought assistance in managing communicable diseases only

Abbreviations: OR, Odds Ratio; CI, Confidence Interval; NCDs, Non-communicable diseases

Negative associations were observed between incomplete healthcare utilization and rural residences (OR:0.8; 95%CI:0.6,0.9) and average monthly income (OR:0.9; 95%CI:0.9,0.9). Negative associations were also found between inaccessibility to healthcare and household size (OR:0.4; 95%CI:0.4,0.5), residence (OR:0.7; 95%CI:0.6,0.8), and income (OR:0.9; 95%CI:0.9,0.9). Inaccessibility to healthcare due to supply-side factors was positively associated with education (OR:1.2; 95%CI:1.02,1.5), poor financial status (OR:1.2; 95%CI:1.02,1.5), and need assistance in managing NCDs (OR:1.2; 95%CI:1.01,1.6); but negatively associated with household size (OR:0.4; 95%CI:0.4,0.5) and income (OR:0.9; 95%CI:0.9,0.9). The odds of healthcare inaccessibility due to demand-side factors were more than two times higher with household size (OR:2.4; 95%CI: 1.9,3.0) but had a negative association with the need for assistance in managing NCDs (OR:0.7; 95%CI: 0.5,0.8).

#### Bangladesh

Immediately after the COVID-19 outbreak, respondents in Bangladesh had more than three times higher odds of experiencing incomplete utilization of healthcare (OR:3.7; 95%CI:1.4,9.4), two times higher odds of experiencing inaccessibility to healthcare (OR:2.0; 95%CI:1.1,3.7), and non-adherence to medication due to supply-side factors (OR:2.3; 95%CI:1.3,4.1) compared to the ‘Pre-COVID phase’. On the contrary, non-adherence to medication due to demand-side factors was 60% less likely (OR:0.4; 95%CI:0.3,0.8) during the ‘Initial phase of COVID-19 outbreak’ compared to the ‘Pre-COVID phase’. After one year of COVID-19 outbreak, participants in Bangladesh were 90% less likely to encounter inaccessibility to healthcare due to demand-side factors (OR:0.1; 95%CI:0.03,0.6).

Participant’s age had two and a half higher odds of experiencing incomplete utilization of healthcare (OR:2.5; 95%CI:1.05,6.2). A large household had 80% less likely to experience non-adherence to medication due to supply-side factors (OR:0.2; 95%CI:0.09,0.7) but three times higher odds of experiencing non-adherence to medication due to demand-side factors (OR:3.0; 95%CI:1.1,8.4). Further, financial status had nearly three times higher odds of experiencing non-adherence to medication due to supply-side factors (OR:2.7; 95%CI:1.003,7.5), but 70% less likely to experience non-adherence to medication due to demand-side factors (OR:0.3; 95%CI:0.1,0.8). Individuals seeking support for NCDs management had nearly four times higher odds of experiencing non-adherence to medication (OR: 3.7; 95%CI: 1.9, 7.3) but were 90% less likely to experience non-adherence to medication due to demand-side factors (OR: 0.1; 95%CI: 0.04, 0.8).

#### India

Respondents in India were 30% more likely to experience non-adherence to medication (OR:1.3; 95%CI:1.1,1.7) in the ‘Initial phase of COVID-19 outbreak’ compared to the ‘Pre-COVID phase’. Following one year of COVID-19 outbreak the participants had nearly three times higher odds of experiencing non-adherence to medication due to supply-side factors (OR:2.7; 95%CI:1.1, 6.5). In the same period, the respondents were about 90% less likely to face inaccessibility to healthcare due to demand-side factors (OR:0.09; 95%CI:0.02, 0.4) and non-adherence to medication due to demand-side factors (OR:0.1; 95%CI:0.06,0.3).

Not participating in income-generating activity had two times higher odds of experiencing incomplete utilization of healthcare (OR:2.0; 95%CI:1.2,3.5). Individuals from larger households (OR: 0.6; 95%CI: 0.4, 0.9) were 40% less likely to experience healthcare inaccessibility, whereas those not engaged in income-generating activities (OR: 1.7; 95%CI: 1.2, 2.4) were 70% more likely to encounter healthcare inaccessibility. Large households were found to be 50% less likely (OR:0.5; 95%CI:0.2,0.9) to be associated with inaccessibility to healthcare due to supply-side factors but 80% more likely (OR:1.8; 95%CI:1.07, 3.2) to be associated with inaccessibility to healthcare due to demand-side factors. Residence (OR:1.6; 95%CI:1.1,2.3) was 60%, and financial status (OR:1.4; 95%CI:1.02,2.0) was 40% more likely to be associated with non-adherence to medication. Non-adherence to medication due to supply-side factors was negatively associated with age (OR:0.3; 95%CI:0.1,0.6), household size (OR:0.3; 95%CI:0.1,0.6), and income-generating activity (OR:0.3; 95%CI:0.1,0.6). Need assistance in managing NCDs had more than twice higher odds (OR:2.3; 95%CI:1.4,3.6) of experiencing non-adherence to medication and non-adherence to medication due to supply-side factors (OR:2.3; 95%CI:1.1,4.6). Age (OR:2.3; 95%CI:1.1,4.8), household size (OR:2.6; 95%CI:1.3,5.2), and income-generating activity (OR:2.2; 95%CI:1.1,4.3) had more than twice higher odds of experiencing non-adherence to medication due to supply-side factors.

Unadjusted models for all three countries are presented in Supplemental Table 3.

### Type of health service use and its change in Afghanistan, Bangladesh, and India

**Fig. 5:**
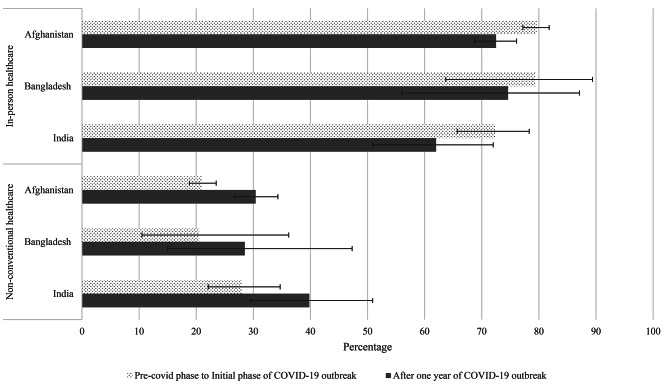
Type of healthcare utilization in Afghanistan, Bangladesh, and India

Utilization of non-conventional health services (e.g., telemedicine and homecare) increased and, use of in-person healthcare decreased in all three countries after one year of COVID-19 outbreak (Fig. 5). However, these changes were not significant at 5% level based on McNemar test (Supplemental Table 4).

### Adjusted association between health service utilization by different types and explanatory variables

After one year of COVID-19 outbreak, respondents in Afghanistan were 30% less likely to use the in-person care (OR:0.7; 95%CI:0.6,0.9) but 40% more likely to use non-conventional care (OR:1.4; 95%CI:1.1,1.8). Income (OR:1.08; 95%CI:1.02,1.1) and household size (OR:1.3; 95%CI:1.06,1.7) were positively associated with utilizing facility-based healthcare among the Afghan respondents (Table 3). Similar significant but negative associations were also observed between utilizing non-conventional health services and income (OR:0.9; 95%CI:0.8,0.9) and household size (OR:0.6; 95%CI:0.5,0.8) (Table 3). Both the participants of Bangladesh (OR:2.9; 95%CI:0.4,20.0) and India (OR:1.6; 95%CI:0.9,2.8) had higher odds of using non-conventional care after one year of COVID-19 outbreak. However, none of the results were statistically significant.

**Table 3:** Multivariate generalized estimating equation model^†^ to examine the association between health service utilization by different types and explanatory variables in Afghanistan, Bangladesh, and India

	Afghanistan		Bangladesh		India	
Variables	In-person care	Non-conventional healthcare	In-person care	Non-conventional healthcare	In-person care	Non-conventional healthcare
	OR (95% CI)	OR (95% CI)	OR (95% CI)	OR (95% CI)	OR (95% CI)	OR (95% CI)
**Environmental factor**						
After one year of COVID-19 outbreak	0.7 (0.6,0.9)*	1.4 (1.1,1.8)*	0.4 (0.09,2.5)	2.9 (0.4,20.0)	0.6 (0.3,1.1)	1.6 (0.9,2.8)
**Pre-disposing factors**						
26 years and above	0.8 (0.6,1.1)	1.1 (0.8,1.4)	1.1 (0.8,4.6)	0.8 (0.2,3.6)	0.8 (0.4,1.4)	1.0 (0.6,1.9)
Male	1.2 (0.8,1.9)	0.8 (0.5,1.2)	-	-	0.7 (0.3,1.5)	1.3 (0.6,2.7)
Less than tertiary education	0.9 (0.7,1.2)	1.0 (0.8,1.3)	3.8 (0.6,22.8)	0.1 (0.02,1.2)	0.9 (0.5,1.7)	1.0 (0.5,1.8)
Large household	1.3 (1.06,1.7)*	0.6 (0.5,0.8)*	1.4 (0.2,10.0)	0.5 (0.08,3.0)	1.1 (0.6,2.1)	0.8 (0.4,1.5)
**Enabling/Disabling factors**						
Rural	1.0 (0.7,1.2)	1.0 (0.7,1.2)	1.2 (0.2,5.8)	0.9 (0.2,3.5)	0.8 (0.5,1.5)	1.0 (0.6,1.8)
Poor financial status	0.9 (0.7,1.2)	0.9 (0.7,1.2)	0.2 (0.02,3.7)	3.8 (0.2,53.5)	0.7 (0.4,1.3)	0.6 (0.2,1.3)
Not participating in any income-generating activity	0.8 (0.6,1.1)	1.1 (0.8,1.4)	0.8 (0.2,3.3)	0.8 (0.2,3.1)	1.1 (0.6,2.2)	1.1 (0.6,2.1)
Income	1.08 (1.02,1.1)*	0.9 (0.8,0.9)*	0.7 (0.4,1.2)	1.3 (0.7,2.5)	1.0 (0.9,1.1)	0.9 (0.8,1.0)
**Need for care factors**						
Need assistance in managing NCDs	0.8 (0.6,1.1)	1.2 (0.9,1.6)	2.5 (0.6,10.4)	0.4 (0.09,2.2)	0.8 (0.4,1.6)	1.2 (0.6,2.3)

*p value < 0.05

^†^All covariates were mutually adjusted

Reference group for Impact of COVID = “Pre-COVID phase to Initial phase of COVID-19 outbreak”, gender = female, age = less than 26 years, education = less than tertiary education, household size = Small household, residence = urban, financial status = better financial status, income generating activity = participated in income-generating activity, and need for care factors = those who sought assistance in managing communicable diseases only

Abbreviations: OR, Odds Ratio; CI, Confidence Interval; NCDs, Non-communicable diseases

Unadjusted models for all three countries are presented in Supplemental Table 5.

## Discussion

The precarious nature of healthcare in South Asia can be traced even before the outbreak of COVID-19 because of their poor resource allocation and weak health system infrastructure. COVID-19 outbreak with bottlenecks of the health systems in South Asia such as health workforce crisis, high out-of-pocket expenditure, and lack of social insurance scheme made the health system too fragile to cope with the sudden shift [21]. The present study examined the effect of the COVID-19 pandemic on healthcare utilization and medication use in three South Asian countries from the perspectives of community members.

It is important to emphasize that most previous studies focused on examining changes in health service utilization during the COVID-19 pandemic at two-time points. However, this study took a more comprehensive approach by observing changes at three distinct time points during and before the pandemic [10]. Furthermore, this study employed advanced statistical analysis by using GEE to assess the impact of COVID-19 through time-varying factors which allowed to provide critical interpretations of the drivers that influenced healthcare utilization. Additionally, this research contributes to promising evidence regarding access to necessary medications during and after COVID-19, where the existing literature is limited. This study adds valuable insights to understand healthcare utilization dynamics during the pandemic. The study suggests that the COVID-19 pandemic had a detrimental effect on the utilization of healthcare in the three South Asian countries. The study observed that immediately after the COVID-19 outbreak, respondents in Bangladesh experienced incomplete utilization of healthcare and inaccessibility to healthcare compared to their similar experience during the ‘pre-COVID phase’, and this observation is identical to the result of other studies conducted in a similar context [11, 31].

Respondents of all three countries were less likely to face inaccessibility to healthcare because of demand-side factors after one year of COVID-19 outbreak, which indicates the linkage between the utilization of healthcare and social resilience toward COVID-19 [42]. Contrary to Bangladesh, respondents in Afghanistan experienced higher incomplete utilization of healthcare before the outbreak of COVID-19. This scenario might indicate that the onset of the impact of COVID-19 on healthcare utilization in Afghanistan appears to have been swift, commencing immediately after information about a cluster of pneumonia cases of unknown origin spread. This early response suggests an atmosphere of uncertainty, observed not only among health professionals providing care but also among patients and their families [43]. Afghan respondents faced inaccessibility to healthcare after one year of COVID-19 outbreak. The overwhelmed healthcare system might explain the scenario. Because at that time, they also faced healthcare inaccessibility due to supply-side factors, and at the same period, Afghanistan experienced the highest number of daily COVID-19 confirmed cases per million (52.26 cases per million) [44]. Though this study did not specifically assess the impact of lockdown measures, it is important to note that lockdowns significantly affect healthcare accessibility in South Asian region by temporarily closing or reducing the capacity of healthcare facilities. These restrictions lead to delays in receiving medical care and create barriers for individuals seeking treatment for non-COVID-19-related health conditions [29, 30].

Incomplete utilization of healthcare was significantly affected by not participating in any income-generating activity in both Afghanistan and India. Among the Afghan respondents, it was found that with the increase in average income, the event of incomplete utilization of healthcare, healthcare inaccessibility, and healthcare inaccessibility due to supply-side factors are less likely to happen. These findings are supported by a systematic review of socioeconomic disparities and COVID-19, based on 52 studies [45]. The study suggested that participants from Afghanistan who belonged to a large household were less likely to experience healthcare inaccessibility. But a reverse result was found when it was inspected at the angle of supply-side factors. A similar observation was observed for India as well. In the South Asian context, family members play an important role in facilitating healthcare access during COVID-19 by providing instrumental, emotional, and financial supports [46]. A study in India during COVID-19 identified that preferred information sources for seeking COVID-19 related information were families and friends [47]. In Afghanistan, awareness of humanitarian aid availability and mobile phones with Subscriber Identity Module (SIM) cards increased healthcare access, and in a large family, there was more chance to have a family member with a SIM card [48]. On the other hand, family members’ or social opinions could delay or avoid the utilization of healthcare [32].

Consistent with time series investigations done in China [49], this study suggests a lack of impact on healthcare utilization in rural areas of Afghanistan and Bangladesh. This study identified that both the participants of India and Bangladesh faced non-adherence to medication use due to supply-side factors during the initial phase of COVID-19 outbreak. These findings could be linked to price changes and medication shortages in Bangladesh and India [50, 51]. However, participants in Afghanistan were more likely to report demand-side factors for their non-adherence to medication. In all three countries, large households were protected from experiencing non-adherence to medication use due to supply-side factors. This indicates the support of family members to ensure medication adherence during COVID-19. The finding was supported by studies conducted in Italy and Portugal [52, 53]. Interestingly, it is an important risk factor of non-adherence to medication due to demand-side factors, which could be linked with fear of spreading COVID-19 infection [54]. In addition, the study found that in Afghanistan and Bangladesh, poor financial status increases the probability of encountering non-adherence to medication use due to supply-side factors, and there were other studies that supported this association [55–57]. Non-adherence to medications has profound implications both for individual health and population health since it can result in disease progression, diminished functional abilities, a decreased quality of life, and an increased utilization of medical resources, such as frequent hospital visits and admissions [58]. Regrettably, the impact of non-adherence to medications could not be assessed due to the lack of availability of relevant data.

Additionally, factors such as the type of medication (e.g., injection versus oral), the number of medications, dosing frequency, the presence of multiple chronic diseases, perceptions of treatment efficacy, concerns about medication costs, and health insurance coverage could potentially influence medication non-adherence [59, 60]. However, present research could not incorporate these factors into the analysis due to data unavailability. The respondents who sought assistance in managing NCDs were more likely to stumble with medication adherence, and this experience was observed for all three countries. A similar experience was observed for patients with NCDs from Portugal, Turkey, and the United Kingdom. However, reasons for non-adherence to medication differ across the countries [53, 61, 62]. Only in Afghanistan the reason for non-adherence to medication could be explained from the angle of supply-side factors. Those who sought support for NCD management in Afghanistan also faced inaccessibility to healthcare because of supply-side factors.

The effect of COVID-19 on NCDs is complex [16]. Physical distancing or quarantine can lead to poor behavioural risk factors such as an unhealthy diet, physical inactivity, tobacco use, and harmful use of alcohol could be an outcome of physical distancing or quarantine [63]. Further, a lockdown situation can lead to disruption of routine medical care and diagnosis, and limited access to pharmacy and primary health care can interrupt the continuity of NCD management. Recent evidence highlighted that reduction in hospital admissions due to acute coronary syndrome led to a rise in out-of-hospital deaths and long term complications following the event [64]. A survey by World Health Organization reported that 48% of countries experienced partial health service disruption among the participants with NCDs [65].

This study implies that there was significant reduction in using in-person healthcare and rise in non-conventional healthcare (telemedicine/homecare) in Afghanistan; it might portray the country’s effort to mitigate the impact of COVID-19 by promoting telemedicine (Afghanistan: TelemedAF) [66] and home healthcare [67]. Using different types of healthcare services in Afghanistan and its relationship with income, raised the question of health equity, a common problem of LMICs during COVID-19 [45]. While this study did not identify a significant rise in the use of non-conventional healthcare in Bangladesh and India. However, the heightened use of non-conventional healthcare after one year of outbreak indicates countries’ efforts to augment health systems by promoting telemedicine and home care [68–70]. In response to emergencies like the COVID-19 outbreak, digital platforms have played a pivotal role in enhancing healthcare access and medication adherence, as reflected in the findings of this study. These platforms facilitate patient self-management, offering services such as telemedicine consultations and digital prescription delivery for extended medication supplies [71, 72]. A study revealed that digital platforms were linked to reduced health system costs, increased productivity for many health services, and overwhelmingly positive patient benefits [73]. Additionally, community patient networks were leveraged to distribute essential medicines efficiently, ensuring continuity of care during crises [74].

This study is limited in its generalizability to other countries of South Asia due to variations in healthcare systems, the differential impact of COVID-19, and distinct sociocultural factors prevalent in each South Asian nation. Moreover, the utilization of data collected through online methods may engender selection bias and response bias, while the retrospective aspect of the ‘Pre-COVID phase’ responses introduces potential limitations arising from recall bias. Common strategies to minimise this recall bias of self-reported data could be the careful selection of the research questions and an appropriate data collection method, such as studying respondents with the onset of COVID-19. Given the secondary nature of the datasets used, the scope of employing such strategies is limited for this study [75]. In this study, the term ‘health services’ was intentionally used as a broad and inclusive category to encompass various aspects of healthcare delivery without specific differentiation between primary care and secondary care or outpatient and inpatient services, as the survey questionnaire was not designed to distinguish among these subcategories.

To the best of our knowledge, this study is among the few studies that analysed the panel data in these three countries, where the influence of COVID-19 on healthcare utilization and medication use were reported from the community perspective. The major limitation of this study is loss-to-follow-up, where Afghanistan, Bangladesh, and India experienced higher dropouts (Supplemental Tables 6, 7 and 8), and this could be internal migrations and displacement because of lockdown and economic reasons. There was significant difference in characteristics between the participants those who participated in both surveys and those who were lost during follow-up (Supplemental Table 9), and it might overestimate or underestimate the result.

Further respondent’s location within the country (such as state or division) was not available, which has a considerable effect on healthcare utilization and medication use, since the number of COVID-19 cases and level of lockdown restriction might differ across the state/division of a country [76]. It would have been valuable to present healthcare utilization and medication use rates specific to individual medical conditions, as these rates can vary significantly from one disease to another. However, this study is constrained by a limited sample size for each medical condition. To overcome this problem, this study aggregated the data that provided healthcare utilization and medication use rates specific to NCDs and provided a more comprehensive view of healthcare utilization and medication use within the broader category. Additionally, it is important to acknowledge that there are additional social and cultural factors, including stigma, religious beliefs, family support, language barriers, traditional healing practices, and cultural perceptions of health, that can profoundly influence healthcare-seeking behaviour [77, 78]. Understanding these influences is vital for enhancing healthcare access and medication adherence within diverse communities of South Asia. Unfortunately, these specific aspects were not covered in the survey data.

## Conclusion

Our analyses shed light on the impacts of the COVID-19 pandemic on healthcare utilization and medication use in three countries of South Asia. Considering the sample size and unavailability of locations of respondents within the country, this result should be viewed as a glimpse of the impact of COVID-19 on healthcare utilization and medication use. Lessons learned from this study will assist researchers and policymakers from South Asia in developing local context-specific solutions to the challenges brought by this pandemic or taking necessary preparation to counteract future pandemics.

### Electronic supplementary material

Below is the link to the electronic supplementary material.

Supplementary Material 1

Supplementary Material 2

Supplementary Material 3

Supplementary Material 4

Supplementary Material 5

Supplementary Material 6

Supplementary Material 7

Supplementary Material 8

Supplementary Material 9

## Data Availability

The datasets are available for public at the website of Institute for Health Metrics and Evaluation (IHME) [https://ghdx.healthdata.org/record/ihme-data/premise-general-population-covid-19-health-services-disruption-survey-2020].
