# Supplementary material for: Patterns and determinants of healthcare utilization and medication use before and during the COVID-19 crisis in Afghanistan, Bangladesh, and India
Source: BMC Health Serv Res. 2024 Apr 3;24:416. doi: 10.1186/s12913-024-10789-4 (PMC10988829; doi:10.1186/s12913-024-10789-4)
Supplement: Supplementary file 1 — Supplementary Material 1 [file 12913_2024_10789_MOESM1_ESM.docx]

Supplemental Table 1 Characteristics of the participants who participated in the survey after one year of COVID-19 outbreak

| **Characteristics** | **Afghanistan^†^**  **n = 1372** | **Bangladesh^‡^**  **n = 59** | **India^⁂^**  **n=238** |
| --- | --- | --- | --- |
|  | Percentage (95% CI)/  Median (IQR) | Percentage (95% CI)/  Median (IQR) | Percentage (95% CI)/  Median (IQR) |
| **Predisposing factors** | | | |
| Age | | | |
| 26 years and above | 32.0 (29.3,34.6) | 45.6 (31.8,60.1) | 36.8 (30.5,43.5) |
| Gender |  |  |  |
| Male | 86.4 (84.1,88.5) | 89.0 (75.9,95.4) | 83.4 (77.4,88.1) |
| Education |  |  |  |
| Less than tertiary education | 66.4 (63.9,68.9) | 53.5 (39.6,66.9) | 46.4 (39.5,53.4) |
| Household size* | | | |
| Large household | 45.1 (42.4,47.8) | 30.7 (20.2,43.6) | 21.4 (16.6,27.0) |
| **Enabling factors** | | | |
| Residence | | | |
| Rural | 37.7 (35.0,40.5) | 40.8 (28.0,55.0) | 37.5 (31.2,44.4) |
| Financial situation* | | | |
| Poor financial situation | 70.3 (67.6,72.9) | 66.7 (51.8,78.8) | 45.9 (38.7,53.4) |
| Income generating activity during February’2021 – May’2021* | | | |
| No | 51.0 (48.3, 53.8) | 37.5 (24.5, 52.6) | 54.1 (47.3,60.7) |
| Average income (USD) in a month during Feb’2021 – May’2021 | 26.9 (103.3) | 60.0 (100.3) | 138.4 (262.8) |
| **Need for care factors** | | | |
| Need assistance in managing NCDs during February’2021 – May’2021 | | | |
| Yes | 71.5 (68.4,74.5) | 70.4 (55.3,82.0) | 68.1 (59.3,75.9) |
| ^†^ Number of missing values for Afghanistan: Gender = 45, Education = 42, Financial situation = 99.  ^‡^ Number of missing values for Bangladesh: Financial situation = 7.  ^⁂^ Number of missing values for India: Gender = 6, Education = 9, Financial situation = 39.  * Variables that are significantly different (*p* value <0.05) across three countries.  Note: Comparisons across three countries were conducted using Kruskal–Wallis test for continuous variables and χ^2^ test for categorical variables.  Abbreviations: CI, Confidence Interval; IQR, Interquartile Range; USD, United States Dollar; NCDs, Non-communicable diseases. | | | |
