# Supplementary material for: Patterns and determinants of healthcare utilization and medication use before and during the COVID-19 crisis in Afghanistan, Bangladesh, and India
Source: BMC Health Serv Res. 2024 Apr 3;24:416. doi: 10.1186/s12913-024-10789-4 (PMC10988829; doi:10.1186/s12913-024-10789-4)
Supplement: Supplementary file 2 — Supplementary Material 2 [file 12913_2024_10789_MOESM2_ESM.docx]

Supplemental Table 2 Changes in healthcare utilization and medication use between ‘Pre-covid phase’, ‘Initial phase of COVID-19 outbreak’, and ‘After one year of COVID-19 outbreak’ in Afghanistan, Bangladesh, and India

| **Variable** | **Difference between ‘Pre-covid phase’ and ‘Initial phase of COVID-19 outbreak’** | | **Difference between ‘Pre-covid phase’ and ‘After one year of COVID-19 outbreak’** | |
| --- | --- | --- | --- | --- |
|  | **McNemar χ^2^** | ***p* value** | **McNemar χ^2^** | ***p* value** |
| **Incomplete utilization of healthcare** | | | | |
| Afghanistan | 16.3 | <0.05 | 12.2 | <0.05 |
| Bangladesh | 3.2 | 0.07 | 0.3 | 0.5 |
| India | 0.7 | 0.4 | 0.1 | 1 |
| **Inaccessibility to healthcare** | | | | |
| Afghanistan | 4.7 | <0.05 | 0.6 | 0.4 |
| Bangladesh | 6.5 | <0.05 | 0.3 | 1 |
| India | 0.1 | 0.8 | 0.3 | 0.6 |
| **Inaccessibility to healthcare due to supply-side factors** | | | | |
| Afghanistan | 0.2 | 0.6 | 3.7 | 0.05 |
| Bangladesh | 1.0 | 1 | 1.0 | 1 |
| India | 0.2 | 0.8 | 0.1 | 1 |
| **Inaccessibility to healthcare due to demand-side factors** | | | | |
| Afghanistan | 0.2 | 0.6 | 24.3 | <0.05 |
| Bangladesh | 1.0 | 1 | 1.0 | 1 |
| India | 0 | 1 | 0.2 | 0.8 |
| **Non-adherence to medication** | | | | |
| Afghanistan | 240.0 | <0.05 | 5.06 | <0.05 |
| Bangladesh | 0.4 | 0.6 | 0.8 | 0.3 |
| India | 13.4 | <0.05 | 0.05 | 1 |
| **Non-adherence to medication due to supply-side factors** | | | | |
| Afghanistan | 1.1 | 0.2 | 0.1 | 0.6 |
| Bangladesh | 3.0 | 0.2 | 1.0 | 1 |
| India | 0.4 | 0.6 | 0 | 1 |
| **Non-adherence to medication due to demand-side factors** | | | | |
| Afghanistan | 7.7 | <0.05 | 0 | 1 |
| Bangladesh | 2.0 | 0.5 | 1.0 | 1 |
| India | 0.3 | 0.7 | 0 | 1 |
| Note: The McNemar test was employed to analyse paired data and observe the change. | | | | |
