# Supplementary material for: Patterns and determinants of healthcare utilization and medication use before and during the COVID-19 crisis in Afghanistan, Bangladesh, and India
Source: BMC Health Serv Res. 2024 Apr 3;24:416. doi: 10.1186/s12913-024-10789-4 (PMC10988829; doi:10.1186/s12913-024-10789-4)
Supplement: Supplementary file 3 — Supplementary Material 3 [file 12913_2024_10789_MOESM3_ESM.docx]

Supplemental Table 3 Univariate generalized estimating equation model to examine the association between predictor variables and healthcare utilization, and use of medication

| Variables | **Incomplete utilization of healthcare** | **Inaccessibility to healthcare** | **Inaccessibility to healthcare due to supply-side factors** | **Inaccessibility to healthcare due to supply-side factors** | **Non-adherence to medication** | **Non-adherence to medication due to supply-side factors** | **Non-adherence to medication due to demand-side factors** |
| --- | --- | --- | --- | --- | --- | --- | --- |
|  | OR (95% CI) | OR (95% CI) | OR (95% CI) | OR (95% CI) | OR (95% CI) | OR (95% CI) | OR (95% CI) |
| **Afghanistan** | | | | | | | |
| **Environmental factor** | | | | | | | |
| Initial phase of COVID-19 outbreak | 0.8 (0.7, 0.9)* | 0.9 (0.8, 0.9)* | 1.1 (0.9, 1.3) | 0.8 (0.7, 1.0) | 1.8 (1.7, 2.0)* | 0.9 (0.8, 1.0) | 1.2 (1.02, 1.4)* |
| After one year of COVID-19 outbreak | 0.7 (0.6, 0.9)* | 1.1 (1.0, 1.3) | 1.3 (1.03, 1.7)* | 0.3 (0.2, 0.4)* | 0.7 (0.6, 0.8)* | 0.7 (0.5, 0.9)* | 1.2 (0.8, 1.7) |
| **Pre-disposing factors** |  |  |  |  |  |  |  |
| 26 years and above | 1.1 (0.9, 1.3) | 1.0 (0.9, 1.2) | 0.8 (0.7, 1.1) | 0.9 (0.7, 1.2) | 0.7 (0.6, 0.8)* | 0.8 (0.6, 1.1) | 1.1 (0.7, 1.5) |
| Male | 0.9 (0.7, 1.1) | 0.7 (0.6, 0.9)* | 1.3 (0.9, 1.7) | 0.7 (0.5, 0.9)* | 1.3 (1.09, 1.6)* | 1.0 (0.7, 1.4) | 0.8 (0.5, 1.4) |
| Less than tertiary education | 1.1 (0.9, 1.3) | 1.1 (1.0, 1.3) | 1.5 (1.2, 1.8) | 0.6 (0.5, 0.8)* | 1.1 (0.9, 1.2) | 1.6 (1.3, 2.0)* | 0.5 (0.4, 0.7)* |
| Large household | 0.9 (0.8, 1.1) | 0.5 (0.4, 0.6)* | 0.3 (0.3, 0.4)* | 2.6 (2.1, 3.3)* | 0.8 (0.7, 0.9)* | 0.2 (0.2, 0.3)* | 4.8 (3.4, 6.7)* |
| **Enabling/disabling factors** |  |  |  |  |  |  |  |
| Rural | 0.9 (0.8, 1.1) | 0.7 (0.6 0.8)* | 1.1 (0.9, 1.4) | 0.8 (0.6, 1.0) | 0.3 (0.2, 0.5)* | 1.2 (0.9, 1.5) | 0.6 (0.5, 0.9)* |
| Poor financial status | 1 (0.9, 1.2) | 0.9 (0.8, 1.0) | 1.3 (1.06, 1.6)* | 0.8 (0.6, 0.9)* | 1.0 (0.9, 1.1) | 1.2 (1.01, 1.6)* | 0.8 (0.6, 1.1) |
| Not participating in an income-generating activity | 1.7 (1.4, 1.9)* | 1.9 (1.7, 2.1)* | 0.8 (0.7, 1.0) | 1.1 (0.9, 1.4) | 0.3 (0.3, 0.4) | 0.5 (0.4, 0.7)* | 1.7 (1.3, 2.2)* |
| Income | 0.9 (0.9, 0.9)* | 0.9 (0.9, 0.9)* | 0.9 (0.9, 0.9)* | 1.05 (1.01, 1.08)* | 1.0 (0.9, 1.09) | 0.9 (0.9, 1.0) | 0.9 (0.9, 1.0) |
| **Need for care factor** |  |  |  |  |  |  |  |
| Need assistance in managing NCDs | 1.2 (1.0, 1.4) | 1.1 (1.02, 1.3)* | 1.5 (1.2, 1.9)* | 0.5 (0.4, 0.7)* | 1.6 (1.4, 1.9)* | 2.1 (1.6, 2.8)* | 0.2 (0.1, 0.3)* |
| **Bangladesh** | | | | | | | |
| **Environmental factor** |  |  |  |  |  |  |  |
| Initial phase of COVID-19 outbreak | 3.6 (1.5, 8.7)* | 2.1 (1.2, 3.7)* | 1.2 (0.5, 2.8) | 0.7 (0.3, 1.7) | 1.0 (0.7, 1.4) | 1.7 (1.1, 2.5)* | 0.6 (0.4, 0.9)* |
| After one year of COVID-19 outbreak | 0.7 (0.1, 3.7) | 1.0 (0.4, 2.5) | 2.4 (0.5, 10.9) | 0.2 (0.04, 1.2) | 1.4 (0.6, 3.3) | 0.6 (0.2, 1.6) | 1.5 (0.5, 4.2) |
| **Pre-disposing factors** |  |  |  |  |  |  |  |
| 26 years and above | 2.8 (1.2, 6.5)* | 1.7 (0.8, 3.5) | 0.5 (0.1, 1.5) | 2.1 (0.7, 6.1) | 1.1 (0.6, 2.1) | 0.9 (0.4, 2.0) | 1.1 (0.5, 2.6) |
| Male | 0.4 (0.1, 2.2) | 0.8 (0.2, 2.5) | - | - | 0.9 (0.3, 3.1) | 1.6 (0.3, 8.5) | 5.5 (0.7, 38.4) |
| Less than tertiary education | 1.0 (0.4, 2.7) | 0.8 (0.4, 1.7) | 1.7 (0.5, 5.9) | 0.6 (0.1, 2.1) | 1.1 (0.6, 2.1) | 0.6 (0.2, 1.7) | 1.4 (0.5, 3.7) |
| Large household | 1.4 (0.6, 3.2) | 1.3 (0.7, 2.6) | 0.7 (0.2, 2.0) | 1.4 (0.5, 3.8) | 0.9 (0.4, 1.7) | 0.3 (0.1, 0.9) | 2.5 (0.9, 6.6) |
| **Enabling/disabling factors** |  |  |  |  |  |  |  |
| Rural | 0.4 (0.1, 1.1) | 0.7 (0.3,1 .3) | 0.6 (0.2, 1.8) | 1.2 (0.4, 3.4) | 1.4 (0.8, 2.6) | 1.4 (0.5, 3.9) | 0.6 (0.2, 1.9) |
| Poor financial status | 1.2 (0.5, 3.0) | 1.3 (0.6, 2.6) | 0.6 (0.2, 1.9) | 1.4 (0.4, 4.3) | 0.6 (0.3, 1.5) | 2.3 (0.9, 5.9) | 0.3 (0.1, 1.0) |
| Not participating in an income-generating activity | 1.2 (0.5, 2.8) | 1.5 (0.8, 2.9) | 0.7 (0.2, 1.9) | 1.4 (0.5, 3.9) | 0.5 (0.3, 0.9)* | 1.0 (0.4, 2.2) | 0.6 (0.2, 1.4) |
| Income | 1.0 (0.8, 1.2) | 0.9 (0.7, 1.0) | 0.9 (0.7, 1.1) | 1.0 (0.8, 1.2) | 1.0 (0.9, 1.1) | 1.0 (0.9, 1.2) | 1.0 (0.8, 1.1) |
| **Need for care factor** |  |  |  |  |  |  |  |
| Need assistance in managing NCDs | 0.7 (0.3, 1.8) | 1.2 (0.5, 2.5) | 2.0 (0.7, 5.8) | 0.4 (0.1, 3.1) | 3.8 (1.9, 7.4)* | 3.7 (0.7, 17.4) | 0.1 (0.04, 0.9)* |
| **India** | | | | | | | |
| **Environmental factor** |  |  |  |  |  |  |  |
| Initial phase of COVID-19 outbreak | 0.8 (0.5, 1.3) | 0.8 (0.6, 1.1) | 1.6 (1.02, 2.6)* | 0.6 (0.3, 0.9)* | 1.3 (1.1, 1.6)* | 0.9 (0.6, 1.3) | 0.9 (0.7, 1.2) |
| After one year of COVID-19 outbreak | 0.7 (0.3, 1.5) | 0.9 (0.5, 1.4) | 2.4 (1.1, 5.4)* | 0.06 (0.01, 0.2)* | 0.9 (0.6, 1.5) | 1.4 (0.8, 2.4) | 0.4 (0.2, 0.8)* |
| **Pre-disposing factors** |  |  |  |  |  |  |  |
| 26 years and above | 0.8 (0.4, 1.4) | 0.8 (0.6, 1.2) | 1.1 (0.6, 1.9) | 0.7 (0.4, 1.2) | 1.1 (0.8, 1.6) | 0.4 (0.2, 0.7)* | 1.7 (0.9, 3.1) |
| Male | 1.1 (0.6, 2.1) | 1.0 (0.7, 1.5) | 1.3 (0.7, 2.3) | 0.6 (0.3, 1.1) | 1.5 (1.03, 2.4)* | 1.8 (0.9, 3.6) | 0.4 (0.2, 1.0) |
| Less than 13 years of education | 1.5 (0.9, 2.4) | 1.2 (0.9, 1.7) | 1.0 (0.6, 1.7) | 0.8 (0.5, 1.4) | 1.2 (0.8, 1.6) | 1.0 (0.6, 1.7) | 1.2 (0.6, 2.1) |
| Large household | 0.7 (0.4, 1.1) | 0.6 (0.4, 0.9)* | 0.4 (0.2, 2.8) | 2.2 (1.3, 3.7) | 0.6 (0.4, 0.9)* | 0.3 (0.1, 0.5)* | 2.7 (1.4, 5.0)* |
| **Enabling/disabling factors** |  |  |  |  |  |  |  |
| Rural | 1.1 (0.7, 1.8) | 1.0 (0.7, 1.3) | 1.3 (0.8, 2.1) | 0.8 (0.5, 1.2) | 1.9 (1.3, 2.6)* | 1.3 (0.8, 2.3) | 0.7 (0.4, 1.2) |
| Poor financial status | 1.5 (0.9, 2.4) | 1.0 (0.7, 1.4) | 0.8 (0.5, 1.3) | 1.2 (0.8, 2.0) | 1.7 (1.2, 2.4)* | 1.5 (0.9, 2.5) | 0.7 (0.4, 1.2) |
| Not participating in an income-generating activity | 1.8 (1.1, 3.0)* | 1.6 (1.2, 2.2) | 0.7 (0.4, 1.1) | 1.3 (0.8, 2.1) | 0.2 (0.2, 0.3)* | 0.4 (0.2, 0.8)* | 1.9 (1.05, 3.7) |
| Income | 0.9 (0.8, 0.9)* | 1.0 (0.9, 1.0) | 0.9 (0.9, 1.0) | 1.0 (0.9, 1.1) | 1.0 (0.9, 1.0) | 0.9 (0.9, 1.0) | 0.9 (0.9, 1.0) |
| **Need for care factor** |  |  |  |  |  |  |  |
| Need assistance in managing NCDs | 1.1 (0.6, 1.8) | 0.9 (0.7, 1.3) | 1.2 (0.7, 2.0) | 0.7 (0.4, 1.2) | 2.7 (1.7, 4.0)* | 2.4 (1.3, 4.4)* | 0.3 (0.1, 0.6)* |
| * *p* value <0.05.  Reference group for Impact of covid = Pre-covid phase, gender = female, age = less than 26 years, education = less than tertiary education, household size = Small household, residence = urban, financial status = better financial status, income generating activity = participated in income-generating activity, and need for care factors = those who sought assistance in managing communicable diseases only.  Abbreviations: OR, Odds Ratio; CI, Confidence Interval; NCDs, Non-communicable diseases. | | | | | | | |
