# Supplementary material for: Patterns and determinants of healthcare utilization and medication use before and during the COVID-19 crisis in Afghanistan, Bangladesh, and India
Source: BMC Health Serv Res. 2024 Apr 3;24:416. doi: 10.1186/s12913-024-10789-4 (PMC10988829; doi:10.1186/s12913-024-10789-4)
Supplement: Supplementary file 4 — Supplementary Material 4 [file 12913_2024_10789_MOESM4_ESM.docx]

Supplemental Table 4 Changes in utilization of healthcare by different types from ‘Pre-covid phase to Initial phase of COVID-19 outbreak’ and ‘After one year of COVID-19 outbreak’ in Afghanistan, Bangladesh, and India

| **Type of health service** | **Difference between ‘Pre-covid phase to Initial phase of COVID-19 outbreak’ and ‘After one year of COVID-19 outbreak’** | |
| --- | --- | --- |
|  | **McNemar χ^2^** | ***p* value** |
| **Afghanistan** |  |  |
| In-person care | 0.06 | 0.9 |
| Non-conventional healthcare service | 1.1 | 0.3 |
| **Bangladesh** |  |  |
| In-person care | 0 | 1 |
| Non-conventional healthcare service | 1.0 | 1.0 |
| **India** |  |  |
| In-person care | 0.6 | 0.6 |
| Non-conventional healthcare service | 0.1 | 1 |
| Note: The McNemar test was employed to analyse paired data and observe the change. | | |
