# Supplementary material for: Patterns and determinants of healthcare utilization and medication use before and during the COVID-19 crisis in Afghanistan, Bangladesh, and India
Source: BMC Health Serv Res. 2024 Apr 3;24:416. doi: 10.1186/s12913-024-10789-4 (PMC10988829; doi:10.1186/s12913-024-10789-4)
Supplement: Supplementary file 5 — Supplementary Material 5 [file 12913_2024_10789_MOESM5_ESM.docx]

Supplemental Table 5 Univariate generalized estimating equation model to examine the association between health service utilization by different types and explanatory variables in Afghanistan, Bangladesh, and India

|  | **Afghanistan** | | **Bangladesh** | | **India** | |
| --- | --- | --- | --- | --- | --- | --- |
| **Variables** | **In-person care** | **Non-conventional healthcare** | **In-person care** | **Non-conventional healthcare** | **In-person care** | **Non-conventional healthcare** |
|  | **OR (95% CI)** | **OR (95% CI)** | **OR (95% CI)** | **OR (95% CI)** | **OR (95% CI)** | **OR (95% CI)** |
| **Environmental factor** |  |  |  |  |  |  |
| After one year of COVID-19 outbreak | 0.7 (0.6,0.9)* | 1.5 (1.2,1.8)* | 0.6 (0.2,1.8) | 1.7 (0.5,5.2) | 0.6 (0.3,1.1) | 1.6 (0.9,2.7) |
| **Pre-disposing factors** |  |  |  |  |  |  |
| 26 years and above | 0.8 (0.6,1.1) | 1.1 (0.8,1.4) | 0.8 (0.2,2.9) | 1.0 (0.3,3.5) | 0.8 (0.5,1.4) | 1.0 (0.6,1.8) |
| Male | 1.3 (0.9,2.0) | 0.7 (0.5,1.1) | - |  | 0.7 (0.3,1.4) | 1.4 (0.7,2.9) |
| Less than tertiary education | 0.9 (0.7,1.1) | 1.09 (0.8,1.3) | 3.7 (1.02, 14.0)* | 0.2 (0.05,0.8)* | 0.8 (0.5,1.4) | 1.1 (0.6,1.9) |
| Large household | 1.4 (1.1, 1.8)* | 0.6 (0.5,0.7)* | 1.8 (0.4,7.6) | 0.5 (0.1,2.1) | 1.4 (0.8,2.6) | 0.6 (0.3,1.2) |
| **Enabling/Disabling factors** |  |  |  |  |  |  |
| Rural | 0.9 (0.7,1.2) | 1.0 (0.8,1.3) | 0.9 (0.2,2.9) | 1.2 (0.3,3.9) | 0.9 (0.5,1.5) | 1.0 (0.6,1.7) |
| Poor financial status | 0.9 (0.7,1.1) | 1.0 (0.8,1.2) | 0.7 (0.1,2.8) | 1.4 (0.3,5.2) | 0.7 (0.4,1.1) | 1.3 (0.8,2.2) |
| Not participating in any income-generating activity | 0.8 (0.6,1.0) | 1.1 (0.8,1.4) | 0.5 (0.1,1.6) | 1.7 (0.5,5.6) | 1.1 (0.6,2.1) | 0.8 (0.4,1.5) |
| Income | 1.08 (1.03,1.1)* | 0.9 (0.8,0.9) | 0.8 (0.6,1.06) | 1.2 (0.9,1.5) | 1.02 (0.9,1.1) | 0.9 (0.8,1.0) |
| **Need for care factors** |  |  |  |  |  |  |
| Need assistance in managing NCDs | 0.7 (0.6,1.01) | 1.4 (1.1,1.8)* | 1.6 (0.4,5.2) | 0.7 (0.2,2.6) | 0.8 (0.4,1.5) | 1.2 (0.7,2.1) |
| **p* value <0.05.  Reference group for Impact of covid = ‘Pre-covid phase to Initial phase of COVID-19 outbreak’, gender = female, age = less than 26 years, education = less than tertiary education, household size = Small household, residence = urban, financial status = better financial status, income-generating activity = participated in income-generating activity, and need for care factors = those who sought assistance in managing communicable diseases only.  Abbreviations: OR, Odds Ratio; CI, Confidence Interval; NCDs, Non-communicable diseases. | | | | | | |
