# Supplementary material for: Patterns and determinants of healthcare utilization and medication use before and during the COVID-19 crisis in Afghanistan, Bangladesh, and India
Source: BMC Health Serv Res. 2024 Apr 3;24:416. doi: 10.1186/s12913-024-10789-4 (PMC10988829; doi:10.1186/s12913-024-10789-4)
Supplement: Supplementary file 6 — Supplementary Material 6 [file 12913_2024_10789_MOESM6_ESM.docx]

Supplemental Table 6 Sample size for outcome variables (healthcare utilization) for Afghanistan, Bangladesh, and India in three different time periods

| **Time period** | **Country** | **Incomplete utilization of healthcare** | **Frequent utilization of healthcare** | **Inaccessibility to healthcare** | **Inaccessibility to healthcare due to supply-side factors** | **Inaccessibility to healthcare due to demand-side factors** |
| --- | --- | --- | --- | --- | --- | --- |
|  |  | **No/Total** | **No/Total** | **No/Total** | **No/Total** | **No/Total** |
| **Pre-covid phase** | **Afghanistan** | 1857/2355 | 862/1857 | 1052/2355 | 716/1052 | 336/1052 |
|  | **Bangladesh** | 86/94 | 24/86 | 28/94 | 14/28 | 14/28 |
|  | **India** | 363/409 | 122/363 | 172/409 | 94/172 | 78/172 |
| **Initial phase of COVID-19 outbreak** | **Afghanistan** | 1806/2189 | 851/1806 | 896/2189 | 638/896 | 258/896 |
|  | **Bangladesh** | 51/70 | 20/51 | 33/70 | 13/33 | 13/33 |
|  | **India** | 286/317 | 101/286 | 123/317 | 81/123 | 42/123 |
| **After one year of COVID-19 outbreak** | **Afghanistan** | 635/760 | 287/635 | 371/760 | 273/371 | 52/371 |
|  | **Bangladesh** | 27/29 | 10/27 | 10/29 | 7/10 | 2/10 |
|  | **India** | 89/97 | 43/89 | 39/97 | 29/39 | 2/39 |
