# Supplementary material for: Patterns and determinants of healthcare utilization and medication use before and during the COVID-19 crisis in Afghanistan, Bangladesh, and India
Source: BMC Health Serv Res. 2024 Apr 3;24:416. doi: 10.1186/s12913-024-10789-4 (PMC10988829; doi:10.1186/s12913-024-10789-4)
Supplement: Supplementary file 7 — Supplementary Material 7 [file 12913_2024_10789_MOESM7_ESM.docx]

Supplemental Table 7 Sample size for outcome variables (medication use) for Afghanistan, Bangladesh, and India in three different time periods

| **Time period** | **Country** | **Non-adherence of medication** | **Non-adherence of medication due to supply-side factors** | **Non-adherence of medication due to demand-side factors** |
| --- | --- | --- | --- | --- |
|  |  | **No/Total** | **No/Total** | **No/Total** |
| **Pre-covid phase** | Afghanistan | 1088/2486 | 886/1088 | 105/1088 |
|  | Bangladesh | 44/127 | 26/44 | 15/44 |
|  | India | 161/447 | 118/161 | 33/161 |
| **Initial phase of COVID-19 outbreak** | Afghanistan | 1479/2486 | 1190/1479 | 159/1484 |
|  | Bangladesh | 47/127 | 37/47 | 9/47 |
|  | India | 197/447 | 143/196 | 34/196 |
| **After one year of COVID-19 outbreak** | Afghanistan | 269/726 | 206/269 | 28/269 |
|  | Bangladesh | 18/37 | 10/18 | 6/18 |
|  | India | 39/95 | 34/39 | 2/39 |
