# Supplementary material for: Patterns and determinants of healthcare utilization and medication use before and during the COVID-19 crisis in Afghanistan, Bangladesh, and India
Source: BMC Health Serv Res. 2024 Apr 3;24:416. doi: 10.1186/s12913-024-10789-4 (PMC10988829; doi:10.1186/s12913-024-10789-4)
Supplement: Supplementary file 8 — Supplementary Material 8 [file 12913_2024_10789_MOESM8_ESM.docx]

Supplemental Table 8 Sample size for outcome variables (utilization of healthcare by different types of health services) for Afghanistan, Bangladesh, and India in two time periods

| **Time period** | **Country** | **In-person care** | **Non-conventional healthcare** |
| --- | --- | --- | --- |
|  |  | **No/Total** | **No/Total** |
| **Pre-covid phase to Initial phase of COVID-19 outbreak** | **Afghanistan** | 947/1191 | 253/1191 |
|  | **Bangladesh** | 29/37 | 8/37 |
|  | **India** | 140/194 | 55/194 |
| **After one year of COVID-19 outbreak** | **Afghanistan** | 470/635 | 184/635 |
|  | **Bangladesh** | 19/27 | 9/27 |
|  | **India** | 56/89 | 35/89 |
