# Supplementary material for: Patterns and determinants of healthcare utilization and medication use before and during the COVID-19 crisis in Afghanistan, Bangladesh, and India
Source: BMC Health Serv Res. 2024 Apr 3;24:416. doi: 10.1186/s12913-024-10789-4 (PMC10988829; doi:10.1186/s12913-024-10789-4)
Supplement: Supplementary file 9 — Supplementary Material 9 [file 12913_2024_10789_MOESM9_ESM.docx]

Supplemental Table 9 Comparison of characteristics of participants who participated in both surveys and those who were lost during follow-up

| Variable | **Afghanistan** | | | **Bangladesh** | | | **India** | | |
| --- | --- | --- | --- | --- | --- | --- | --- | --- | --- |
|  | **Participated in both surveys**  **(n = 1372)** | **Lost to follow-up**  **(n = 2464)** | ***p* value** | **Participated in both surveys**  **(n = 59)** | **Lost to follow-up**  **(n = 141)** | ***p* value** | **Participated in both surveys**  **(n = 238)** | **Lost to follow-up**  **(n = 752)** | ***p* value** |
| **Age, n (%)** | | | | | | | | | |
| Less than 26 years | 1041 (75.9) | 1947 (79.3) | < 0.05 | 36 (61.0) | 96 (68.0) | 0.3 | 165 (69.3) | 524 (69.7) | 0.9 |
| 26 years and above | 329 (24.0) | 507 (20.7) |  | 23 (39.0) | 45 (31.9) |  | 73 (30.7) | 228 (30.3) |  |
| **Gender, n (%)** | | | | | | | | | |
| Female | 182 (13.6) | 283 (12.0) | 0.1 | 6 (10.5) | 8 (5.7) | 0.2 | 38 (16.6) | 200 (26.8) | < 0.05 |
| Male | 1153 (86.4) | 2074 (88.0) |  | 51 (89.5) | 133 (94.3) |  | 191 (83.4) | 546 (73.1) |  |
| **Education, n (%)** | | | | | | | | | |
| Tertiary education or higher | 689 (51.1) | 1074 (44.3) | < 0.05 | 38 (65.5) | 102 (73.9) | 0.2 | 142 (60.4) | 477 (64.3) | 0.2 |
| Less than tertiary education | 658 (48.9) | 1351 (55.7) |  | 20 (34.5) | 36 (26.1) |  | 93 (39.6) | 265 (35.7) |  |
| **Household size, n (%)** | | | | | | | | | |
| Small household | 617 (47.0) | 1225 (52.5) | < 0.05 | 36 (61.0) | 78 (55.3) | 0.4 | 179 (75.2) | 492 (65.5) | < 0.05 |
| Large household | 696 (53.0) | 1106 (47.5) |  | 23 (39.0) | 63 (44.7) |  | 59 (24.8) | 259 (34.5) |  |
| **Residence, n (%)** |  |  |  |  |  |  |  |  |  |
| Urban | 927 (67.6) | 1612 (65.4) | 0.1 | 38 (64.4) | 86 (61.0) | 0.6 | 127 (53.3) | 451 (60.0) | 0.07 |
| Rural | 445 (32.4) | 852 (34.6) |  | 21 (35.6) | 55 (39.0) |  | 111 (46.6) | 301 (40.0) |  |
| **Financial situation, n (%)** | | | | | | | | | |
| Better financial situation | 461 (33.6) | 816 (33.1) | 0.7 | 15 (25.4) | 47 (33.3) | 0.2 | 109 (45.8) | 432 (57.5) | < 0.05 |
| Poor financial situation | 911 (66.4) | 1648 (66.9) |  | 44 (74.6) | 94 (66.7) |  | 129 (54.2) | 320 (42.5) |  |
| **Income generating activity during ‘Pre-covid phase’, n (%)** | | | | | | | | | |
| Yes | 659 (48.0) | 1290 (52.3) | < 0.05 | 36 (61.0) | 91 (64.5) | 0.6 | 113 (47.4) | 406 (54.0) | 0.08 |
| No | 713 (52.0) | 1174 (47.6) |  | 23 (39.0) | 50 (35.5) |  | 125 (52.5) | 346 (46.0) |  |
| **Income generating activity during ‘Initial phase of COVID-19 outbreak’, n (%)** | | | | | | | | | |
| Yes | 809 (59.0) | 1616 (65.6) | < 0.05 | 33 (55.9) | 71 (50.4) | 0.4 | 138 (58.0) | 426 (56.6) | 0.7 |
| No | 563 (41.0) | 848 (34.4) |  | 26 (44.1) | 70 (49.6) |  | 100 (42.0) | 326 (43.4) |  |
| Average income (USD) in a month during ‘Pre covid phase’, Median (IQR) | 16.4 (88.5) | 12.5 (83.5) | 0.1 | 60.0 (118.0) | 71.8 (165.2) | 0.1 | 139.3 (352.3) | 142.0 (340.2) | 0.4 |
| Average income (USD) in a month during ‘Initial phase of COVID-19 outbreak’, Median (IQR) | 14.1 (78.9) | 8.9 (78.9) | 0.7 | 24.5 (113.0) | 59.8 (116.9) | 0.5 | 54.0 (238.7) | 67.3 (244.6) | 0.4 |
| **Need assistance in managing NCDs during ‘Pre-covid phase’, n (%)** | | | | | | | | | |
| No | 255 (24.7) | 482 (24.7) | 0.9 | 12 (30.0) | 35 (33.3) | 0.7 | 30 (23.4) | 153 (35.1) | < 0.05 |
| Yes | 777 (75.3) | 1469 (75.3) |  | 28 (70.0) | 70 (66.7) |  | 98 (76.6) | 283 (64.9) |  |
| **Need assistance in managing NCDs during ‘Initial phase of COVID-19 outbreak’, n (%)** | | | | | | | | | |
| No | 267 (26.1) | 472 (24.7) | 0.3 | 11 (28.2) | 35 (35.0) | 0.4 | 30 (23.4) | 136 (34.5) | < 0.05 |
| Yes | 757 (73.9) | 1443 (75.3) |  | 28 (71.8) | 65 (65.0) |  | 98 (76.6) | 258 (65.5) |  |
| Note: To determine the demographic difference between those who participated in both surveys and those who were lost during follow-up, χ^2^ test was done for categorical variables and Mann-Whitney U test was done for continuous variables.  Abbreviations: IQR, Interquartile Range; USD, United States Dollar; NCDs, Non-communicable diseases. | | | | | | | | | |
